# Single image mixed dehazing method based on numerical iterative model and DehazeNet

**DOI:** 10.1371/journal.pone.0254664

**Published:** 2021-07-30

**Authors:** Wenjiang Jiao, Xingwu Jia, Yuetong Liu, Qun Jiang, Ziyi Sun

**Affiliations:** 1 School of Software, Shandong University, Jinan, China; 2 Vehicle Management Office, Shandong Provincial Public Security Department, Jinan, China; 3 School of Computer Science and Technology, Shandong University of Finance and Economics, Jinan, China; Manipal University, INDIA

## Abstract

As one of the most common adverse weather phenomena, haze has caused detrimental effects on many computer vision systems. To eliminate the effect of haze, in the field of image processing, image dehazing has been studied intensively, and many advanced dehazing algorithms have been proposed. Physical model-based and deep learning-based methods are two competitive methods for single image dehazing, but it is still a challenging problem to achieve fidelity and effectively dehazing simultaneously in real hazy scenes. In this work, a mixed iterative model is proposed, which combines a physical model-based method with a learning-based method to restore high-quality clear images, and it has good performance in maintaining natural attributes and completely removing haze. Unlike previous studies, we first divide the image into different regions according to the density of haze to accurately calculate the atmospheric light for restoring haze-free images. Then, dark channel prior and DehazeNet are used to jointly estimate the transmission to promote the final clear haze-free image that is more similar to the real scene. Finally, a numerical iterative strategy is employed to further optimize the atmospheric light and transmission. Extensive experiments demonstrate that our method outperforms existing state-of-the-art methods on synthetic datasets and real-world datasets. Moreover, to indicate the universality of the proposed method, we further apply it to the remote sensing datasets, which can also produce visually satisfactory results.

## Introduction

Due to the absorption and scattering of suspended particles in the atmosphere, images taken in haze, fog, and smoke have low contrast and poor visibility. Hazy images can severely affect the performance of surveillance systems, remote sensing imaging, and computer vision tasks that depend on image quality. The single-image defogging method can recover a haze-free image from a low-quality foggy scene. According to the physical process of hazy image formation [1], a hazy image is described as:

$$\begin{array}{c} I(x)=J(x)t(x)+A(1-t(x)), \end{array}$$ (1)

where *x* is the pixel position. *I* denotes the hazy image, and *J* is the clear image. *t* represents the scene transmission, and *A* represents the atmospheric light. The process of image dehazing aims to recover *J* from *I*, so *t* and *A* need to be estimated, which is a challenging task.

Based on statistical knowledge of fog-free images [2–6], the traditional prior-based method can estimate the atmospheric light and transmission in the atmospheric scattering model. To restore a haze-free image, Fattal [2] first estimated the transmission map by assuming that the scene transmission was unrelated to surface coloring. Zhu et al. [4] estimated the depth information in an image by establishing a linear model and then restored the fog-free image. Berman et al. [5] introduced a global algorithm based on a nonlocal path prior to calculating the transmission of each pixel and then obtained the fog-free image according to the physical model. Fattal [7] found that pixels of images ordinarily exhibit one-dimensional distributions in RGB color space, called color-lines. They deduced a local formation model to explain the color-lines in the hazy scene, and recovered scene transmission according to the color-line and origin offset. Liu et al. [8] proposed a transmission adaptive regularized image recovery method for high-quality single image dehazing. Considering that the amplification effect of artifacts depends on scene transmission, a transmission adaptive regularized recovery approach based on non-local total variation (NLTV) was presented, which can simultaneously suppress visual artifacts and retain image details in the final dehazing result. Based on the observation of clear outdoor images, He et al. [6] introduced a novel haze removal method for an image based on the dark channel prior (DCP), which restores clear images by estimating the transmission. The DCP shows good performance in image defogging, so a series of approaches have been presented to further improve the dehazing effect. [9–16]. Based on the DCP, [13] modified the atmospheric veil and the transmission to remove haze from remote sensing images. Pan et al. [15] introduced a constant into the atmospheric scattering model to remove haze, which was based on the view that the average intensity of dark channels in remote sensing images is low but not close to zero. Jiang et al. [16] introduced an experience-based single-image defogging method, which obtained the haze thickness maps of all bands from the dark channel. Taking into account the uneven distribution of haze thickness and the complexity of the light source, we presented a numerical iterative dehazing algorithm based on the DCP in our previous work [17]. The numerical iterative dehazing method can maintain image details and color fidelity, but the dehazed images still have some fog residue. The above methods rely on certain prior conditions, and when the priors are violated, will lead to inaccurate estimates, so these methods have limited applicability.

With advances in deep learning, many data-driven methods have been presented for single-image dehazing [18–35]. Li et al. [19] presented an all-in-one dehazing network to estimate one variable that is integrated by atmospheric light and transmission. Ren et al. [20] introduced a gated fusion network for restoring fog-free images, which integrates three confidence maps obtained using different physical models. Li et al. [21] presented a defogging model to directly restore fog-free images by utilizing a conditional generative adversarial network. Based on an attention mechanism, Liu et al. [23] introduced GridNet [36] into single-image defogging and introduced a multiscale defogging network. Shao et al. [24] presented a domain adaptation paradigm consisting of an image translation module and two image dehazing modules. The method utilizes the images before and after translation to train networks with a consistency constraint, which further improves the adaptability of the domain. Hong et al. [25] proposed a knowledge-distill dehazing network based on heterogeneous task imitation. By reconstructing the image and designing a spatial-weighted channel attention residual block, the method can better reconstruct the features to restore the haze-free image. Song et al. [26] introduced a deep convolutional neural network to generate the disparity and clear images simultaneously from a hazy stereo image pair. To learn the optimal fusion of depth-related features, a novel encoder-decoder architecture was presented, which extends the core idea of attention mechanism to the simultaneous stereo matching and dehazing. Recently, the problem of irregular and nonuniform distribution of haze in remote sensing images has been solved. Gu et al. [27] presented a single remote sensing image dehazing method that directly produced fog-free images with a prior-based dense attentive dehazing network. Hu et al. [28] presented an unsupervised dehazing method for high-resolution remote sensing images. These learning-based methods can directly restore the fog-free image, but they deviate from the physical model and cannot effectively preserve the physical properties of the image. In contrast to the above methods of directly recovering the fog-free image, Cai et al. [32] presented an end-to-end dehazing network (DehazeNet), which can generate the transmission map and then restore the clear image based on the physical model. Ren et al. [33] presented a multiscale network to compute coarse-to-fine transmission. Zhang and Patel [34] used a densely connected encoder-decoder network and U-Net [37] to estimate the transmission map and atmospheric light, respectively. Jiang et al. [35] introduced a remote sensing image dehazing method utilizing a multiscale residual convolutional neural network (MRCNN). These methods can effectively eliminate the haze effect in the image, but they are trained on synthetic foggy images, so the effectiveness of these methods in processing real-world images using learned intermediate parameters is limited.

Considering that physical model-based methods have poor performance in thorough haze removal, learning-based methods have a limited generalization ability in natural scenes. To effectively remove haze from real foggy scenes, we propose a mixed iterative model that uses the DCP and DehazeNet to jointly estimate the transmission. The DCP is based on the atmospheric scattering model, and DehazeNet is data-driven, so the proposed method can preserve the natural attributes of the image and has universal applicability in achieving an effective defogging effect. Due to the uneven fog density in the images, such as remote sensing images and regular natural hazy images, we adopt local atmospheric light instead of global atmospheric light. The experimental results indicate that the presented method has a good defogging effect in fog removal.

The main contributions of our work are as follows:

1. A mixed numerical iterative framework is presented that uses both a physical model-based method and a deep learning-based method for image defogging.
2. We utilize the DCP and DehazeNet to jointly estimate the transmission, which can retain the physical characteristics of the image and learn the distribution of the fog to perform accurate image restoration.
3. We compare the presented method with other state-of-the-art algorithms on synthetic hazy images and natural foggy images. The results of the experiments show that the presented algorithm has good performance. The ablation study further proves the effectiveness of the presented approach.

## Related work

### Single image numerical iterative dehazing method

The DCP [6] is based on the significant observation that the majority of the local area of an outdoor fog-free image (non-sky area) covers some very low pixels approaching 0 in at least one color channel. The dark channel *J*^*dark*^ of a fog-free image can be expressed as:

$$\begin{array}{c} J^{dark}\left(x\right)=\min_{c\in \{r,g,b\}}\left(\min_{y\in \Omega (x)}J^{c}\left(y\right)\right), \end{array}$$ (2)

where *J* denotes the fog-free image, *J*^*c*^ represents a color channel in *J*, and Ω(*x*) denotes a local block whose center is *x*. Utilizing this prior knowledge, [6] estimates the intermediate parameters *A* and *t*(*x*) to reconstruct the clear image according to the atmospheric scattering model. Based on the DCP, we introduce a single-image numerical iterative dehazing method for single-image defogging [17], which can effectively remove the fog from hazy images, preserve the detail of the image and maintain fidelity. This method first divides the region, then estimates the local physical features, and finally recovers the scene iteratively.

#### Region division

Considering that the distribution of fog in an image is not always uniform, the foggy image is divided into different fog density regions by using the affinity propagation (AP) [38] cluster algorithm before defogging. AP is an adaptive clustering algorithm. To represent the fog concentration, we choose the visibility and contrast of the input image as the clustering features. After the three steps of clustering feature selection, correlation matrix calculation and initialization, and matrix update and sample determination, the foggy image is divided into different fog density regions.

#### Local physical features estimation

To reflect the differences in atmospheric light between regions and prevent halo phenomena from emerging in the restored images, we adopt the method proposed in [6] to calculate the local atmospheric light instead of the global atmospheric light. Then, we calculate the transmission based on the DCP. The rough transmission is estimated as:

$$\begin{array}{c} t_{rough}=1-\omega \;\min_{c}\left(\min_{y\in \Omega (x)}\left(\frac{I^{c}\left(y\right)}{A^{c}}\right)\right). \end{array}$$ (3)

In fact, the transmission is not always constant within a local block. The thickness of fog in the distance in a hazy image will exceed that of haze near the camera lens. Hence, the farther a pixel is from the camera, the lower the transmission. Accordingly, we allow slightly different estimated transmission in a local patch. To solve this problem, we refine the rough transmission. The refined formula of each pixel can be described as:

$$\begin{array}{c} t_{ori}\left(i,j\right)=t_{rough}\left(i,j\right)\left(1-0.001\left(D\left(i,j\right)-D_{min}\right)\right), \end{array}$$ (4)

where *t*_*ori*_ denotes the adjusted transmission and is used as the initial value for numerical iterative defogging. *D* = *min*(*I*^*c*^) denotes the minimum value of the three channels of the image block, and *D*_*min*_ denotes the minimum value of *D*.

#### Iteration for scene recovery

After region division and local physical feature estimation, an iterative model is introduced to recover a high-quality and fog-free image. The initial conditions of iterative dehazing can be defined as follows:

$$\begin{array}{c} \left\{ \begin{array}{c} J_{0}=I,\\ t_{0}=t_{ori}, \end{array}\right. \end{array}$$ (5)

where *J*_0_ is the hazy image and *t*_0_ denotes the initial transmission. $A_{0}^{m}\left(m=1,2,\ldots \right)$ is the initial local atmospheric light, which is computed from separate haze density areas of *J*_0_. Then, a numerical iterative dehazing model is constructed according to Eqs (1) and (5), which is expressed as:

$$\begin{array}{c} \left\{ \begin{array}{c} J_{n-1}=J_{n}t_{n-1}+\left(1-t_{n-1}\right)A_{n-1}^{m}\\ t_{n}\left(x\right)=1-\omega \;\min_{c}\left(\min_{y\in \Omega (x)}\left(\frac{J_{n}^{c}}{A_{n}^{m}}\right)\right) \end{array}n=1,2,\ldots ,\right. \end{array}$$ (6)

where *J*_*n*_ represents the *n*-th approximation of the fog-free image. $A_{n}^{m}$ is the *n*-th iteration of local atmospheric light estimated from *J*_*n*_. $A_{n}^{m}$ and *t*_*n*_ denote the approximation of the *n*th iteration calculated from *J*_*n*_.

In the first iteration, we obtain the defogging image *J*_1_ in light of the initial conditions and iteration formula. $A_{1}^{m}$ and *t*_1_ are also obtained at this point. After the first iteration, the dehazing image *J*_1_ is taken as a hazy image for the second iteration, and $A_{2}^{m}$, *t*_2_ and *J*_2_ are obtained after the second iteration. The iterative process is repeated until image *J*_*n*_ becomes a haze-free image and the iteration termination condition is satisfied.

### DehazeNet

The key to dehazing is to calculate a transmission map for the input foggy scene. Cai et al. [32] presented a convolutional neural network-based architecture named DehazeNet, which calculates the transmission and then uses a physical model to recover a fog-free image from a foggy image input.

DehazeNet is made up of four components: feature extraction, multiscale mapping, a local extremum, and nonlinear regression. The Maxout unit [39] is introduced in the first layer of DehazeNet, which is a feed-forward nonlinear activation function. By maximizing the pixel level of *k* affine feature maps, the Maxout unit generates a new feature map, which contains the features related to fog. In the second layer, parallel convolutional operations are used. The same number of convolution kernels of different sizes are employed to carry out convolution operations on the input features to perform multiscale feature extraction. To preserve the resolution of the restored image, the third layer performs a dense local extremum operation on each feature map obtained from the second layer. For the fourth layer, a novel linear unit, the bilateral rectified linear unit (BReLU), is introduced to maintain bilateral restraint and local linearity. These four layers are connected in series to form a trainable end-to-end system based on a CNN, in which the filters and biases related to the convolutional layer are the network parameters that need to be learned.

Note that the convolutional neural network-based DehazeNet is data-driven and trained using paired data of synthetic hazy images and haze-free counterparts. The transmission learned from synthetic data is different from that of natural hazy images. Moreover, DehazeNet regards atmospheric light as a global constant. It is particularly unsuitable for some multisource images and results in the dehazed images suffering color and information loss. Based on local physical properties, the numerical iterative defogging method uses mathematical thinking to calculate the parameters of the physical model iteratively and obtains the parameters from the natural hazy image; it can retain the physical features of the image well and recover the natural color. However, this method relies on prior knowledge, so it has limited universal applicability. In this work, we present an iterative dehazing model based on joint transmission estimation. The transmissions estimated by the numerical iterative dehazing method and DehazeNet are effectively fused. In this way, the clear image restored by our model can effectively remove the fog and maintain fidelity.

## Proposed method

The single-image numerical iterative dehazing method we proposed in [17] is dependent on local physical features; it can remove most of the haze in images and restore appropriate brightness and color levels while maintaining the physical characteristics. DehazeNet uses a deep network to calculate the transmission, and it can effectively remove the fog by relying on paired synthetic data. Therefore, we present a fusion-based method by fusing the transmissions obtained from the physical model-based approach and data-driven method, which can effectively eliminate the fog and preserve the details of the image.

This section introduces the details of the presented approach. First, we outline the presented single-image iterative dehazing method, which consists of three steps: estimation of the local atmospheric light, calculation of transmission, and iterative optimization. Second, for the key steps in our method, we introduce the estimation of transmission in detail. Then, the termination conditions of iterative optimization are described. Finally, we analyze the loss function used in the proposed method.

### Method overview

**Fig 1:**
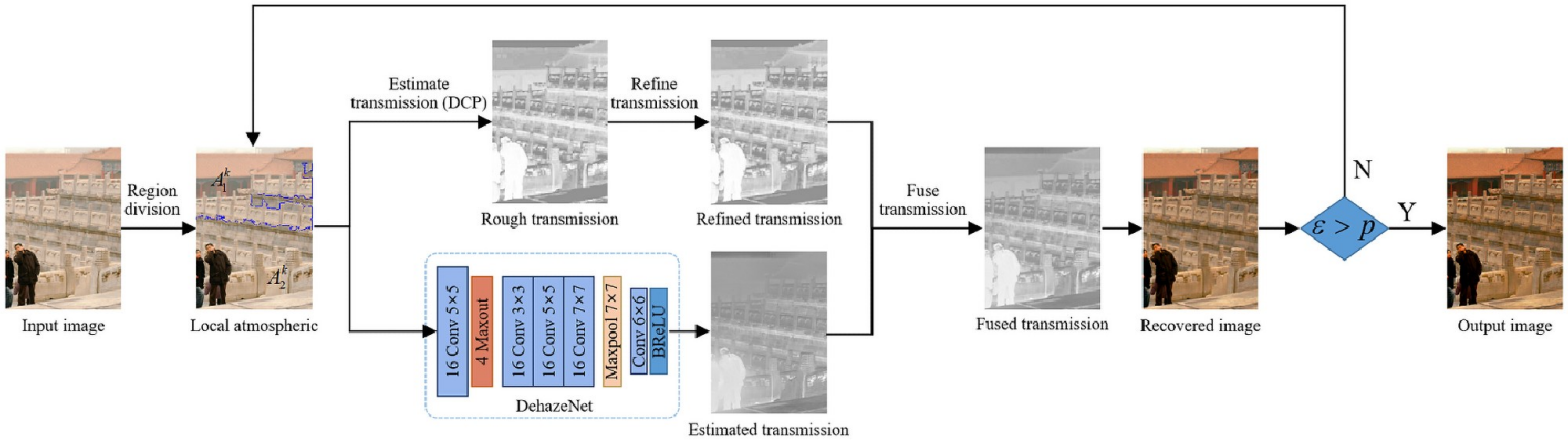
The architecture of the proposed image dehazing algorithm.

Given a hazy image, the presented method aims to produce a fog-free image. The workflow is shown in Fig 1. First, without considering the prior information of the number of clusters, the foggy image is adaptively divided into areas with different fog densities using the AP algorithm. The local atmospheric light is estimated for each haze density area based on the atmospheric scattering model. Then, a physical model-based approach and a data-driven method are adopted to estimate the transmission. The joint transmission is produced by fusing the transmission obtained using the DCP-based method and DehazeNet. Next, we optimize the recovered image iteratively according to the iteration termination condition. Finally, a fog-free image is produced that fits the iteration termination condition and can effectively remove the fog.

The presented approach first trains DehazeNet with paired synthetic images to obtain a pretrained model. Then, the single-image numerical iterative defogging method is adopted to obtain the local atmospheric data and a new, refined transmission. The refined transmission is fused with the medium transmission obtained by the network. The joint transmission and local atmospheric data are used to recover the haze-free image based on the atmospheric scattering model. Finally, according to the iterated termination condition, the recovered image is re-input as the hazy image for iterative defogging. In each iteration, we fine-tune the whole network using the above pretrained model and produce the final dehazing output.

### Estimation of joint transmission

The atmospheric transmittance is expressed by the transmittance *t* (0 < *t* < 1). When *t* is close to 0, the image has low visibility. The estimation of the presented joint transmission consists of two parts: the DCP-based estimation method and the data-driven estimation method.

#### DCP-based estimation method

For a transmission *t*, we first obtain a rough transmission based on the DCP, which assumes that the transmission in a local patch is constant. In fact, the transmission in a local block is not always constant; it alters with the change in fog density in the image. Thus, a refined transmission is introduced, which is defined as:

$$\begin{array}{c} \left\{ \begin{array}{c} J_{k}=J_{k}t_{k-1}+\left(1-t_{k-1}\right)A_{k-1}^{m}\\ t_{k1}\left(x\right)=1-\omega \;\min_{c}\left(\min_{y\in \Omega (x)}\left(\frac{J_{k}^{c}}{A_{k}^{m}}\right)\right) \end{array}k=1,2,\ldots ,\right. \end{array}$$ (7)

where *k* represents the number of iterations. The refined transmission improves the accuracy of the transmission, which ensures realistic color levels in the dehazed images. *t*_*k*,1_(*x*) represents the transmission calculated by the DCP in the *k*th iteration.

#### Data-driven estimation method

The data-driven estimation method learns the mapping between foggy images and their related transmission maps. DehazeNet is made up of cascaded convolutional and pooling layers, and some of these layers use appropriate nonlinear activation functions. After four sequential layers, we obtained an estimation of medium transmission.

The layer of feature extraction is defined as follows:

$$\begin{array}{c} F_{1}^{i}\left(x\right)=\max_{j\in [1,k]}g^{i,j}\left(x\right),g^{i,j}=W_{1}^{i,j}*I+B_{1}^{i,j}, \end{array}$$ (8)

where $W_{1}={\left\{W_{1}^{i,j}\right\}}_{\left(i,j)=(1,1\right)}^{\left(n_{1},k\right)}$ and $B_{1}={\left\{B_{1}^{\left(i,j\right)}\right\}}_{\left(i,j)=(1,1\right)}^{\left(n_{1},k\right)}$ denote the filters and the biases, respectively, and * indicates the convolution operation. *I* is the input image, and after the first layer, we obtain *n*_1_ output feature maps. In this layer, the Maxout unit maps each of the *kn*_1_-dimensional vectors into an *n*_1_-dimensional vector and captures the fog-related features through automatic learning.

For the multiscale mapping, parallel convolutional operations are selected in the second layer. The output can be written as:

$$\begin{array}{c} F_{2}^{i}=W_{2}^{\left\lceil i/3\rceil ,(i/3\right)}*F_{1}+B_{2}^{\left\lceil i/3\rceil ,(i/3\right)}, \end{array}$$ (9)

where $W_{2}={\left\{W_{2}^{p,q}\right\}}_{\left(p,q)=(1,1\right)}^{\left(3,n_{2}/3\right)}$ and $B_{2}={\left\{B_{2}^{\left(p,q\right)}\right\}}_{\left(p,q)=(1,1\right)}^{\left(3,n_{2}/3\right)}$ contain *n*_2_ pairs of parameters that are divided into three groups. *n*_2_ represents the output dimension in the multiscale mapping. *i* ∈ [1, *n*_2_] is the index of the output features.

The third layer is a local extremum option, which is intensively applied to each feature map pixel. It can maintain the resolution for image restoration. It is defined as:

$$\begin{array}{c} F_{3}^{i}\left(x\right)=\max_{y\in \Omega }F_{2}^{i}\left(y\right), \end{array}$$ (10)

where Ω(*x*) is an *f*_3_ × *f*_3_ neighborhood whose center is *x*. The output dimension of the local extremum operation is *n*_3_ = *n*_2_.

The last layer is nonlinear regression, and the BReLU activation function is employed in this layer. In the fourth layer, the feature map is defined as:

$$\begin{array}{c} F_{4}=\min\left(t_{max},\max\left(t_{min},W_{4}*F_{3}+B_{4}\right)\right) \end{array}$$ (11)

where $W_{4}=\left\{W_{4}\right\}$ includes a filter of size *n*_3_ × *f*_4_ × *f*_4_, $B_{4}=\left\{B_{4}\right\}$ includes a bias, and *t*_*max*,*min*_ denotes the marginal value of the BReLU. In accordance with (11), the gradient of this activation function is as follows:

$$\begin{array}{c} \frac{\partial F_{4}\left(x\right)}{\partial F_{3}}=\{\begin{array}{cc} \frac{\partial F_{4}\left(x\right)}{\partial F_{3}}, & t_{min}\leq F_{4}\left(x\right)\leq t_{max}\\ 0, & otherwise \end{array} \end{array}$$ (12)

According to the above four layers, the transmission estimated by DehazeNet *t*_*k*2_ can be obtained. The transmission *t*_*k*2_ estimated by the deep network and *t*_*k*1_ estimated by the physical model are effectively fused. Then, the joint transmission is produced in each iteration for haze-free image recovery, which can be described as:

$$\begin{array}{c} t_{k}=\lambda_{1}t_{k1}+\lambda_{2}t_{k2}, \end{array}$$ (13)

where *λ*_1_ and *λ*_2_ denote the weights of each transmission.

### Iterative optimization

The threshold of the iteration termination criterion is set to the average brightness of the training sample *ε*. The percentage of dark pixels of the recovered image is computed as *p* at the end of each iteration. When *ε* > *p*, iteration should terminate. Otherwise, the recovered image will go through the next loop of the estimation of local atmospheric light and transmission until *ε* > *p*.

### Loss function

DehazeNet employs the mean squared error (MSE) as the loss function, which minimizes the difference between the transmission of the foggy training image and the corresponding fog-free image. The MSE loss is expressed as follows:

$$\begin{array}{c} L\left(\Theta \right)=\frac{1}{N}\sum_{i=1}^{N}{∥F\left(I_{i}^{P};\Theta \right)-t_{i}∥}^{2} \end{array}$$ (14)

where $\Theta =\{W_{1},W_{2},W_{4},B_{1},B_{2},B_{4}\}$ are network parameters. F maps the relationship between the RGB value and the transmission. $I_{i}^{P}$ is the *i*-th training patch. *t*_*i*_ is the ground-truth medium transmission.

## Experimental results

The effectiveness of the presented dehazing model is evaluated by comparison with four advanced prior-based algorithms (DCP [6], CAP [4], NLD [5], and Wang et al.’s method [17]) and five learning-based approaches (MSCNN [33], DehazeNet [32], AOD-net [19], GFN [20], and CycleGAN [22]) in this section. On synthetic datasets and real-world foggy images, we compare the presented approach with other methods. The metrics peak signal-to-noise ratio (PSNR), structural similarity index measure (SSIM) and information entropy are adopted to quantitatively evaluate the experimental results. To prove the contribution of this paper, we conduct an ablation study to analyze the presented method.

### Experimental data

This paper uses the RESIDE dataset [40] to synthesize the training data. The RESIDE dataset utilizes a large number of data sources, including indoor and outdoor scene images (ITS and OTS, respectively). We selected 4000 random fog-free images from ITS and OTS to create the training dataset. Then, 1,000 images were selected from each of these two datasets as the fine-tuned dataset. For each image, we randomly chose 10 transmissions *t* ∈ (0,1) uniformly to generate 10 foggy images. Thus, there are 100,000 hazy images and corresponding fog-free images in total that are adopted as the training data for DehazeNet.

We made qualitative and quantitative comparisons with other competitive methods on both natural and remote sensing images. The HazeRD dataset [41] contains 75 synthesized hazy images with real haze conditions. The remote sensing images were derived from publicly available databases. The NWPU VHR-10 dataset [42, 43] is a 10-level geographic remote sensing dataset for the detection of space objects, which has 800 images consisting of 650 images containing a target and 150 background images. The NWPU-RESISC45 dataset [44] is a remote sensing image scene classification dataset. The RSOD Dataset [45, 46] and Dataset for Object Detection in Aerial Images (DOTA) [47] are two datasets for remote sensing image object detection. These images cover many scenes and different objects and contain different haze densities.

### Implementation details

The experiment in this article is implemented on a computer with an Intel(R) Core(TM) i5-9400F CPU @2.90 GHz, 16 GB of RAM, and an NVIDIA GTX 2080 Ti GPU. The learning rate of DehazeNet is set to 5*e*-3, and it decays by half every 10 epochs. DehazeNet is trained for 50 epochs with a batch size of 16 on the training dataset. The weights of transmission fusion are set to *λ*_1_ = 0.5 and *λ*_2_ = 0.5. Finally, we fine-tune the network utilizing the pretrained model on a fine-tuned dataset.

### Evaluations on synthetic hazy images

**Fig 2:**
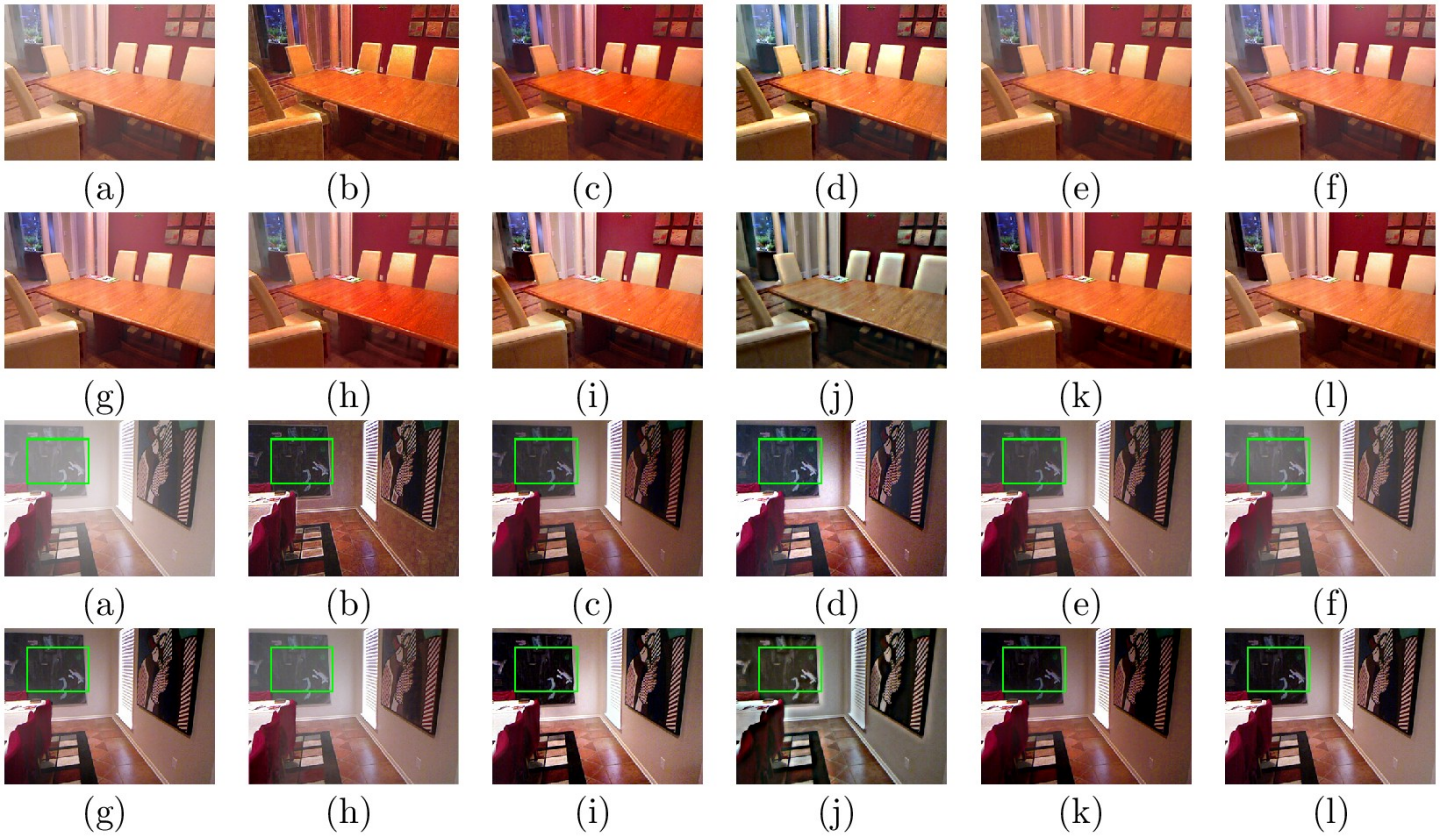
Comparative results of indoor hazy images in SOTS. (a)Hazy (b)DCP [6] (c)CAP [4] (d)NLD [5] (e)Wang [17] (f)MSCNN [33] (g)DehazeNet [32] (h)AOD-net [19] (i)GFN [20] (j)CycleGAN [22] (k)Ours (l)Ground truth.

**Fig 3:**
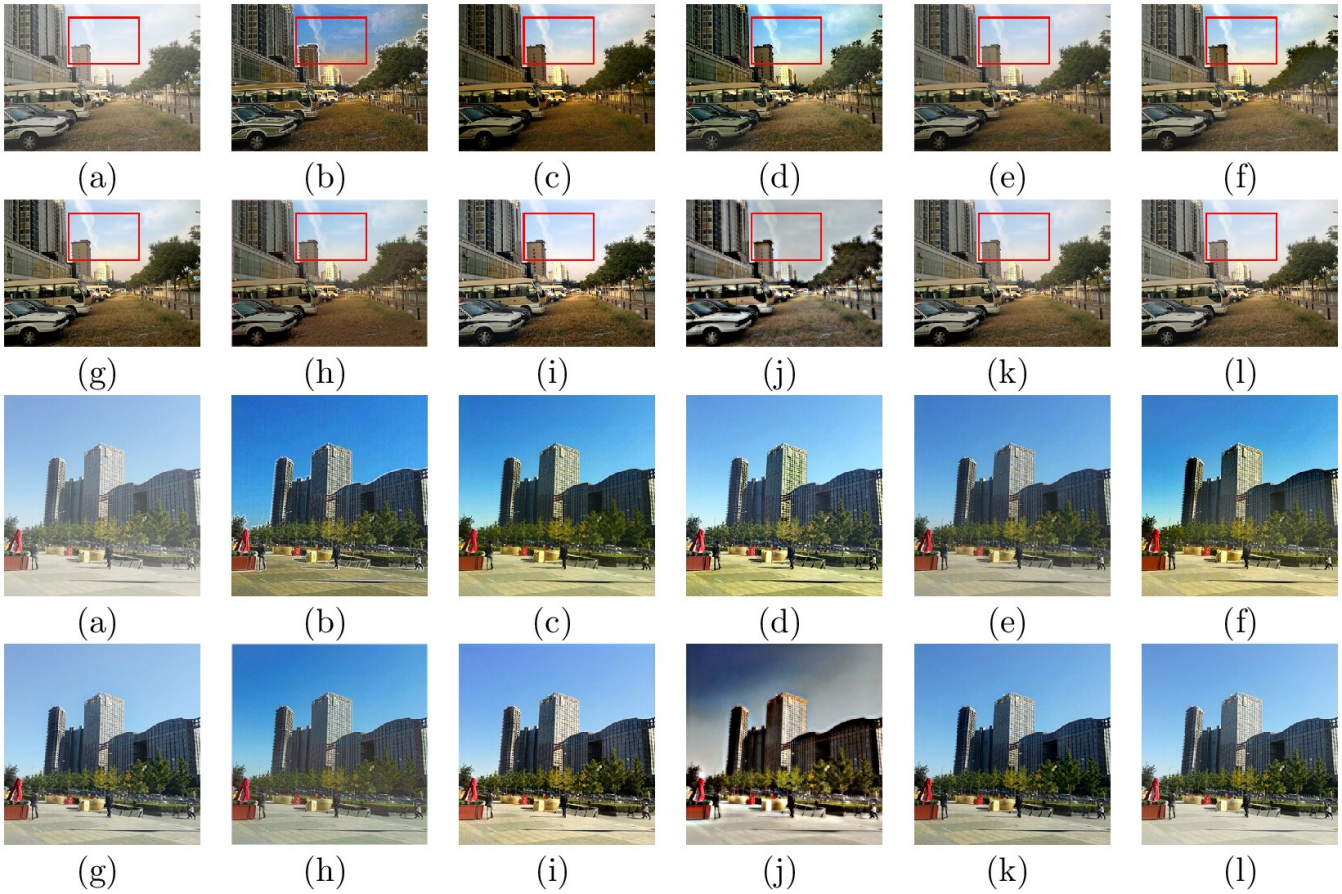
Comparative results of outdoor hazy images in SOTS. (a)Hazy (b)DCP [6] (c)CAP [4] (d)NLD [5] (e)Wang [17] (f)MSCNN [33] (g)DehazeNet [32] (h)AOD-net [19] (i)GFN [20] (j)CycleGAN [22] (k)Ours (l)Ground truth.

**Fig 4:**
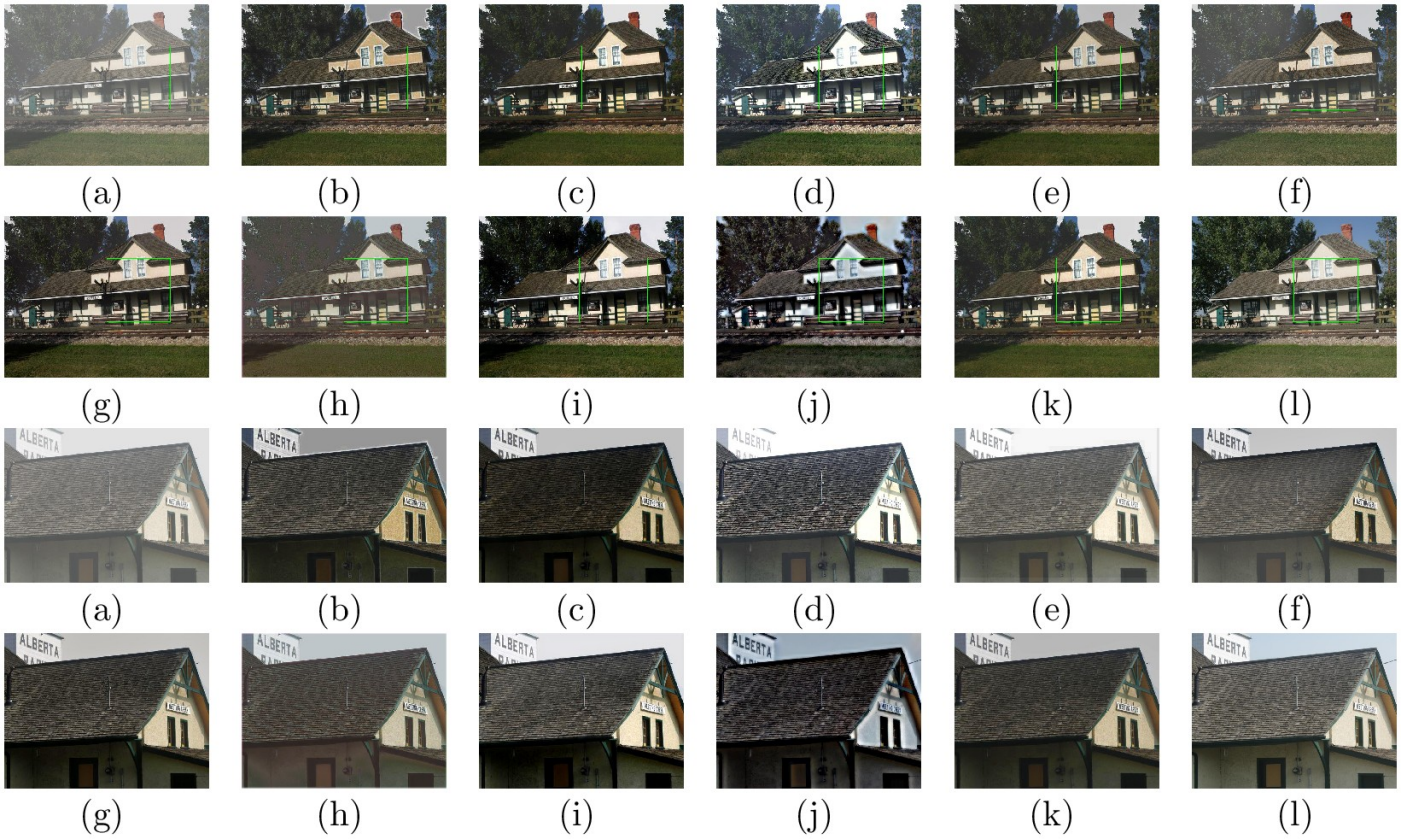
Comparative results of hazy images in HazeRD. (a)Hazy (b)DCP [6] (c)CAP [4] (d)NLD [5] (e)Wang [17] (f)MSCNN [33] (g)DehazeNet [32] (h)AOD-net [19] (i)GFN [20] (j)CycleGAN [22] (k)Ours (l)Ground truth.

We tested the proposed method on synthetic hazy images, and the experiment was performed on the test set SOTS in the RESIDE dataset and HazeRD dataset. Figs 2 and 3 show the dehazing results on indoor, outdoor hazy images in SOTS. Fig 4 shows the comparative results of hazy images in HazeRD. For traditional methods based on the physical model, DCP, CAP, NLD, and Wang et al.’s method all retain some haze in the recovered results, as shown in the second images of (b), (c), (d), and (e) in Figs 2 and 4. Moreover, there are dark areas in the recovered results of DCP and CAP, and the results of NLD are brighter than the ground truth, as shown in Figs 2 and 4(d). For the deep learning-based approaches, MSCNN and AOD-net have limited performance in thorough defogging, as shown in Fig 2(f) and 2(h). DehazeNet, GFN, and CycleGAN can effectively eliminate the haze in the image. However, DehazeNet is poor in color restoration, such as in the second image in Fig 3(g). The image brightness after GFN restoration is significantly brighter, as shown in Figs 2 and 3(i). The dehazed result of CycleGAN has artifacts and unrealistic color tones, as shown in Figs 2 and 3(j). By contrast, the proposed method can effectively eliminate most of the fog and is visually closer to the ground truth, with realistic colors on hazy images in SOTS dataset and HazeRD dataset.

Tables 1 and 2 show the objective results of the presented approach and other comparison approaches of indoor and outdoor hazy images on SOTS test sets. Table 3 lists the quantitative evaluations on the HazeRD dataset. The comparison indicators include PSNR, SSIM, and information entropy. Tables 1–3 prove that the presented method achieves advanced performance on these evaluation matrices, which also indicates the effectiveness of the presented algorithm.

**Table 1:** Quantitative evaluations on indoor hazy SOTS images.

Indoor	PSNR	SSIM	Entropy
DCP [6]	18.1829	0.7826	15.7955
CAP [4]	19.0675	0.8181	15.3750
NLD [5]	17.2671	0.7779	15.4918
Wang [12]	19.9538	0.8334	15.2894
MSCNN [33]	17.1292	0.7958	15.6681
DehazeNet [32]	21.3466	0.8610	15.4705
AOD-net [19]	17.9396	0.8036	15.4294
GFN [20]	22.3200	0.8800	15.8971
CycleGAN [22]	17.2665	0.8017	16.0036
Ours	**22.7583**	**0.8904**	**16.0541**

**Table 2:** Quantitative evaluations on outdoor hazy SOTS images.

Outdoor	PSNR	SSIM	Entropy
DCP [6]	17.0249	0.8442	14.3123
CAP [4]	18.4932	0.7982	13.5870
NLD [5]	18.4747	0.8539	14.5206
Wang [12]	20.9868	0.8724	13.7865
MSCNN [33]	19.6167	0.8791	14.2133
DehazeNet [32]	22.7936	0.8955	13.9069
AOD-net [19]	19.7743	0.8833	14.3812
GFN [20]	21.4900	0.8380	14.8464
CycleGAN [22]	18.0553	0.8523	14.7659
Ours	**23.3952**	**0.9012**	**14.8960**

**Table 3:** Quantitative evaluations on the HazeRD dataset.

	PSNR	SSIM	Entropy
DCP [6]	14.64	0.7761	9.7475
CAP [4]	14.15	0.7434	9.7184
NLD [5]	14.58	0.8100	11.6886
Wang [12]	15.34	0.8163	11.3965
MSCNN [33]	15.62	0.8179	10.0242
DehazeNet [32]	15.30	0.7859	9.8385
AOD-net [19]	15.64	0.8014	10.5736
GFN [20]	13.73	0.6686	11.8655
CycleGAN [22]	15.54	0.7857	12.5179
Ours	**15.78**	**0.8204**	**12.6386**

### Evaluations on real-world hazy images

**Fig 5:**
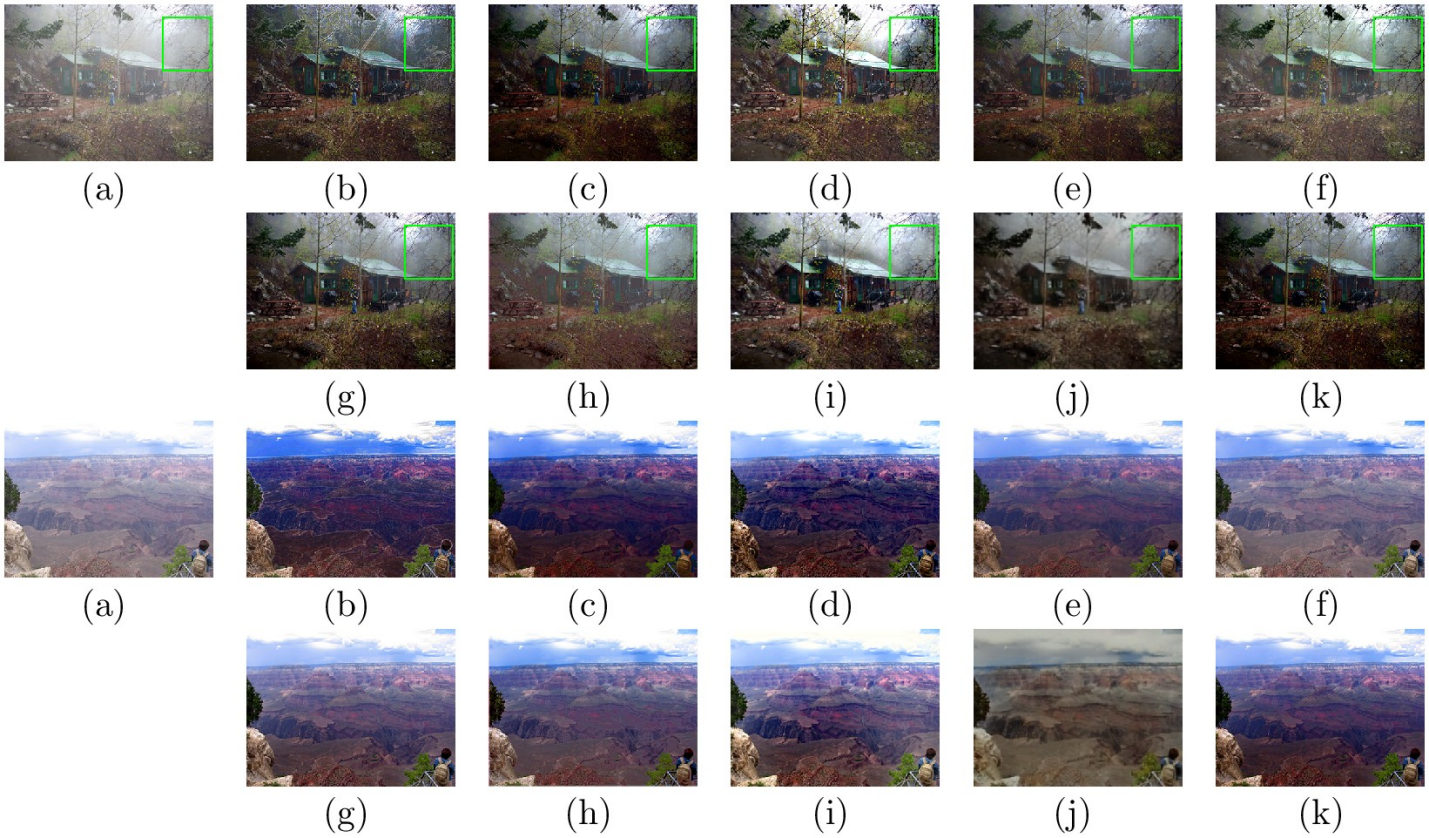
Comparative results of hazy images in HazeRD. (a)Hazy (b)DCP [6] (c)CAP [4] (d)NLD [5] (e)Wang [17] (f)MSCNN [33] (g)DehazeNet [32] (h)AOD-net [19] (i)GFN [20] (j)CycleGAN [22] (k)Ours (l)Ground truth.

**Fig 6:**
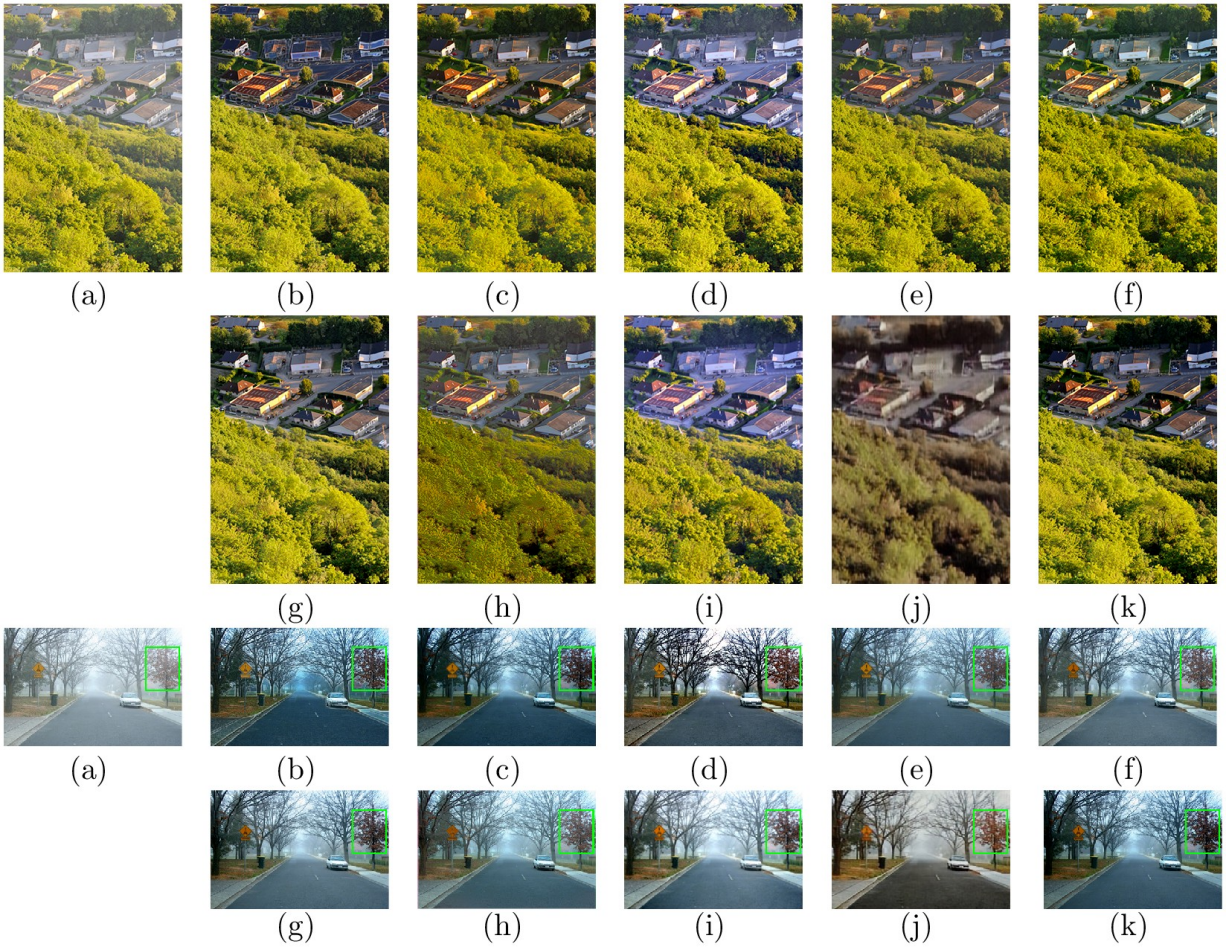
Comparative results on real-world hazy images. (a)Hazy (b)DCP [6] (c)CAP [4] (d)NLD [5] (e)Wang [17] (f)MSCNN [33] (g)DehazeNet [32] (h)AOD-net [19] (i)GFN [20] (j)CycleGAN [22] (k)Ours.

**Fig 7:**
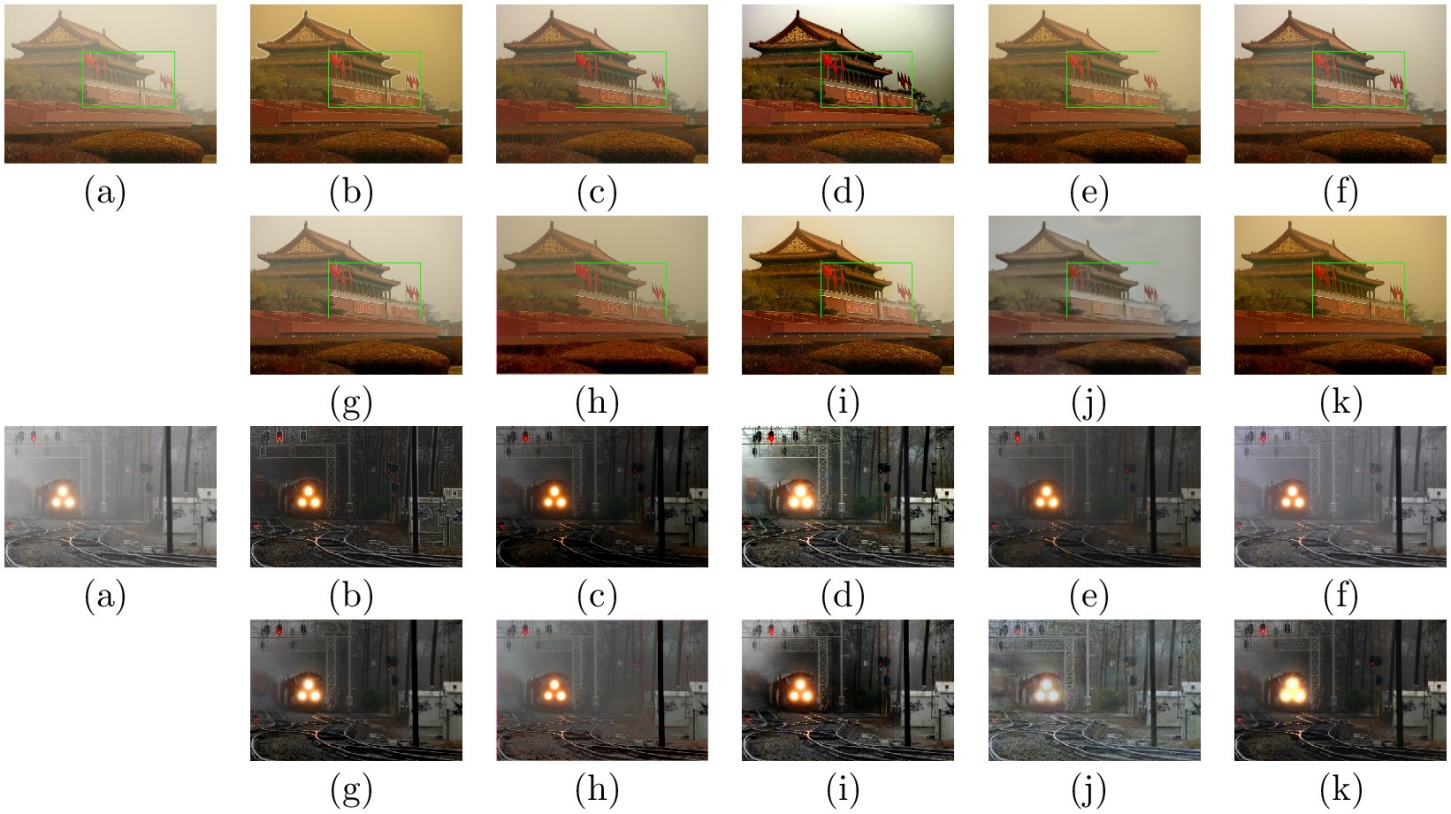
Comparative results on real-world hazy images. (a)Hazy (b)DCP [6] (c)CAP [4] (d)NLD [5] (e)Wang [17] (f)MSCNN [33] (g)DehazeNet [32] (h)AOD-net [19] (i)GFN [20] (j)CycleGAN [22] (k)Ours.

To validate the practicability of the presented model, we compared the visual effect of the presented algorithm with natural hazy image processing using other state-of-the-art technologies. Figs 5 and 6 display the recovered results of all comparison methods on natural foggy images. Fig 7 shows the comparisons on real-world dusty and dense haze images. DCP, CAP, NLD, and Wang et al.’s approach, which are based on the physical model, can effectively process natural hazy images. However, the DCP and CAP results appear too dark in some areas, such as in Figs 5 and 6(b) and 6(c). The local area of the image restored by NLD is too bright, which causes some loss of detail, as shown in Fig 5(d). There is still a layer of mist in the recovered results of the strategy of Wang et al., as shown in Figs 5 and 7(e). Regarding the learning-based models, MSCNN, DehazeNet, and AOD-net have limited defogging effects on natural scenes, and a color shift occurs in the defogging results of AODnet, as shown in Figs 6 and 7 (h). GFN has a good effect in removing most of the fog, but the brightness of the recovered images increases, as shown in Figs 5 and 6(i). CycleGAN suffers color distortion and detail loss, as shown in Figs 5 and 6(j). By comparison, our method generates the highest-fidelity dehazed results on the thin and dense fog images, which can recover results with clear contours and rich colors while reducing the loss of details.

### Evaluations on remote sensing hazy images

**Fig 8:**
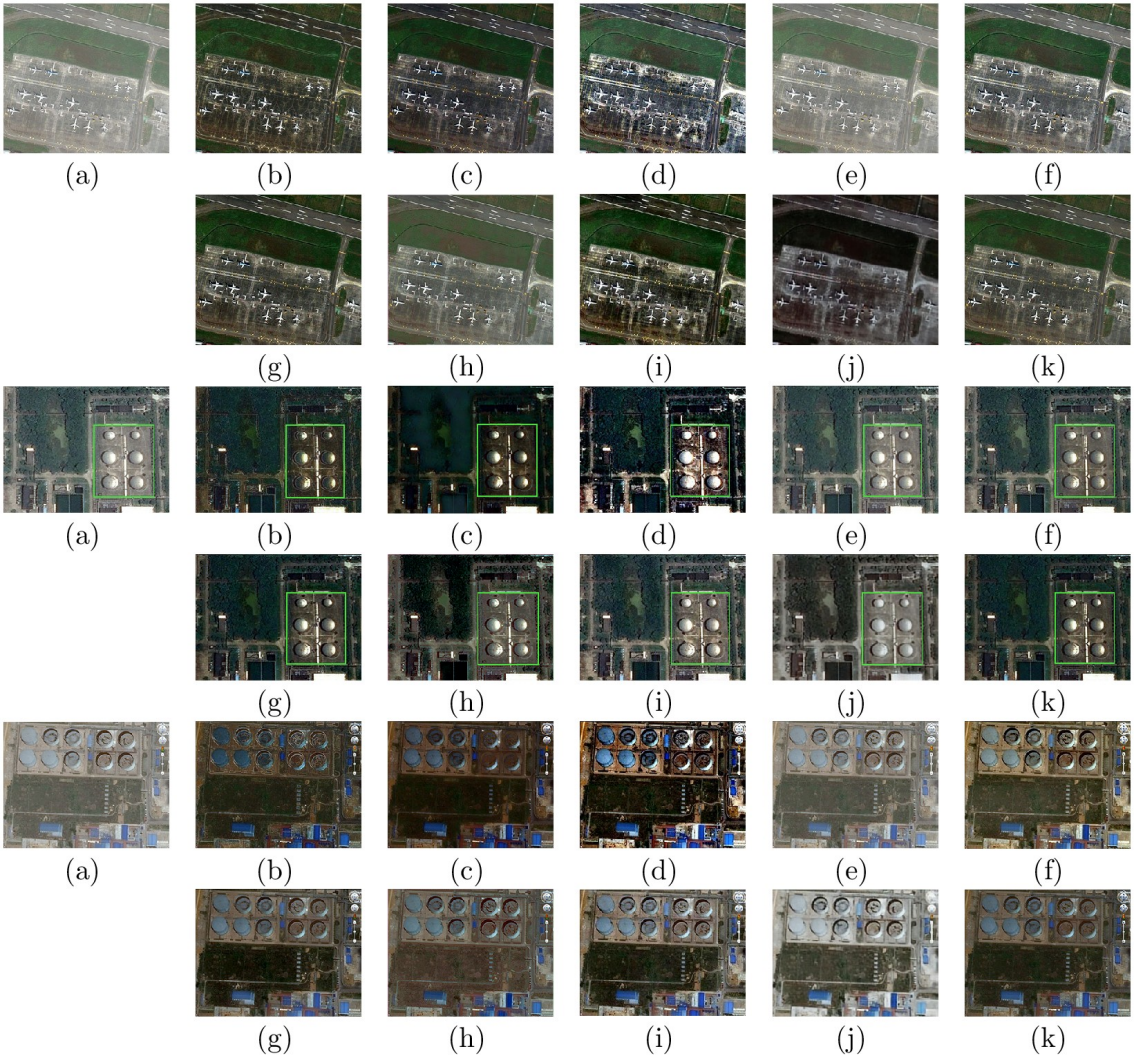
Comparative results of remote sensing hazy images in NWPU VHR-10 dataset [42, 43] and RSOD dataset [45, 46]. (a)Hazy (b)DCP [6] (c)CAP [4] (d)NLD [5] (e)Wang [17] (f)MSCNN [33] (g)DehazeNet [32] (h)AOD-net [19] (i)GFN [20] (j)CycleGAN [22] (k)Ours.

**Fig 9:**
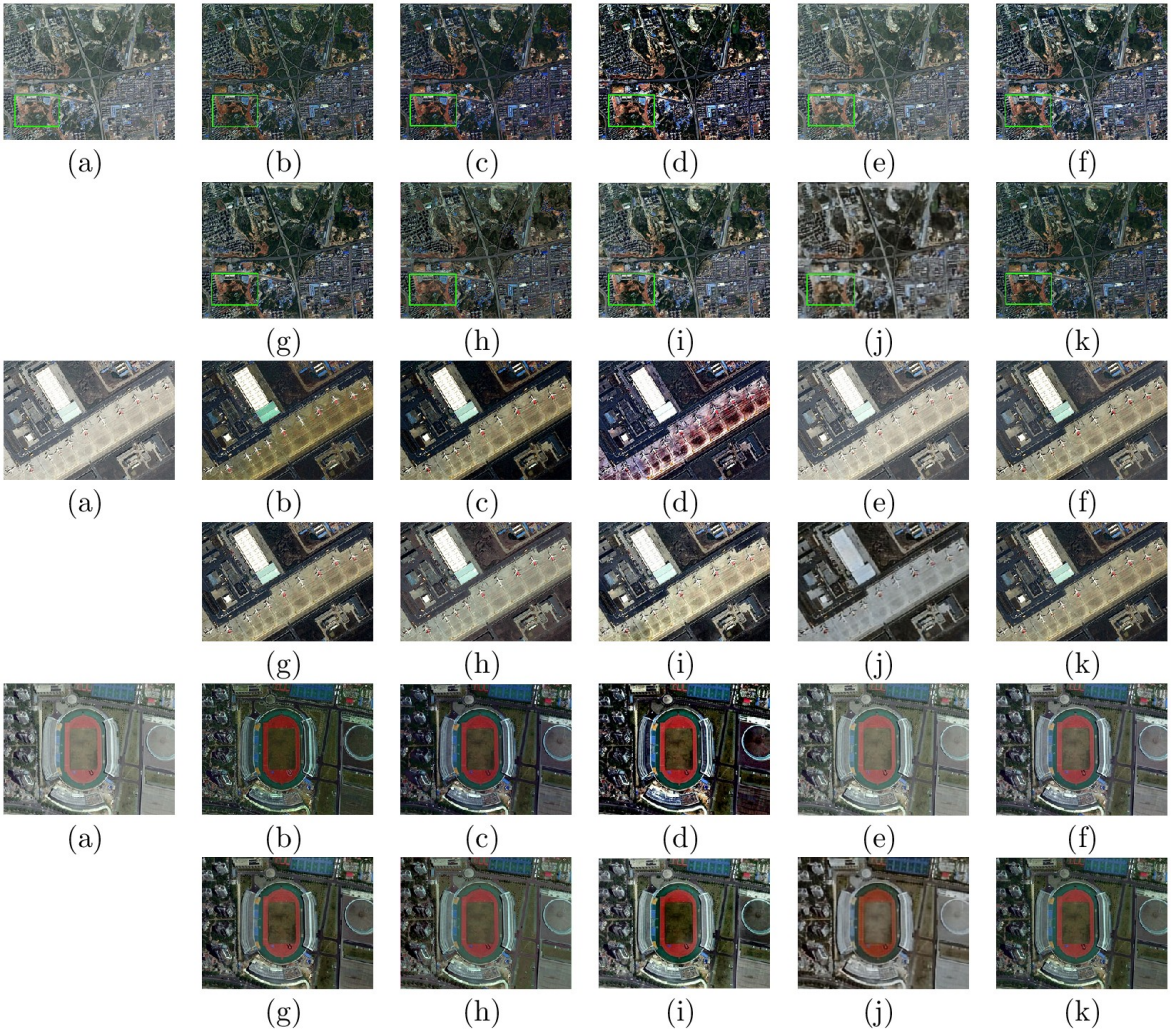
Comparative results on remote sensing hazy images in NWPU-RESISC45 dataset [44] and DOTA dataset [47]. (a)Hazy (b)DCP [6] (c)CAP [4] (d)NLD [5] (e)Wang [17] (f)MSCNN [33] (g)DehazeNet [32] (h)AOD-net [19] (i)GFN [20] (j)CycleGAN [22] (k)Ours.

To further verify the universal applicability of the presented approach, we also conducted experiments on remote sensing data. Figs 8 and 9 show the dehazing results on six different visual scenes, which include forests, rivers, buildings, airports and ports. For the traditional physical model-based methods, DCP, CAP, NLD, and Wang et al.’s method all retain some haze in the recovered result, such as the second image of (b), (c), (d), and (e) in Fig 8. Moreover, there are some dark areas in the DCP and CAP results, such as in Fig 9(a) and 9(b). The NLD results have unrealistic color tones, such as in Figs 8(d) and 9(d). In some cases, the MSCNN and AOD-net methods cannot eliminate the haze effect completely, such as in Fig 9(f) and 9(h). DehazeNet, GFN and CycleGAN can effectively eliminate haze in remote sensing images. DehazeNet is poor in color restoration, such as in the second image in Fig 9(g). After GFN restoration, the brightness of the image clearly increases, as shown in Figs 8(i) and 9(i). The dehazing results of CycleGAN have artifacts and unrealistic color tones, as shown in Figs 8(j) and 9(j). In comparison, our method can effectively remove most of the fog in the remote sensing image and is visually closer to the natural color.

Table 4 shows the information entropy scores of our method and other methods. Our method has the best entropy scores in the NWPU VHR-10 [42, 43], RSOD [45, 46] and DOTA datasets [47]. Although the performance of our method on the NWPU-RESISC45 dataset [44] is slightly poor, it is superior to the other methods in subjective effect. The average result of our method on the four databases is the best, which indicates that our method is more competitive than the other methods.

**Table 4:** Information entropy evaluations on each hazy remote sensing dataset.

Dataset	NWPU VHR-10 [42, 43]	RSOD [45, 46]	NWPU-RESISC45 [44]	DOTA [47]	avgEntropy
DCP [6]	13.7789	15.1023	14.9031	13.3641	14.2871
CAP [4]	14.0156	14.6841	14.7288	13.2972	14.1814
NLD [5]	14.0328	14.2453	15.1367	13.826	14.3102
Wang [12]	13.7231	14.2433	14.3241	13.2249	13.8789
MSCNN [33]	14.1338	14.5234	14.6818	13.7069	14.2610
DehazeNet [32]	13.6643	14.5754	14.9891	13.3013	14.1325
AOD-net [19]	13.5627	14.931	**15.1204**	11.2527	13.7122
GFN [20]	13.5244	14.3791	15.0366	13.7563	14.1741
CycleGAN [22]	12.1814	14.1638	13.2388	13.5851	13.5423
Ours	**14.1352**	**14.7281**	**14.9575**	**13.8295**	**14.4126**

### Ablation study

**Fig 10:**
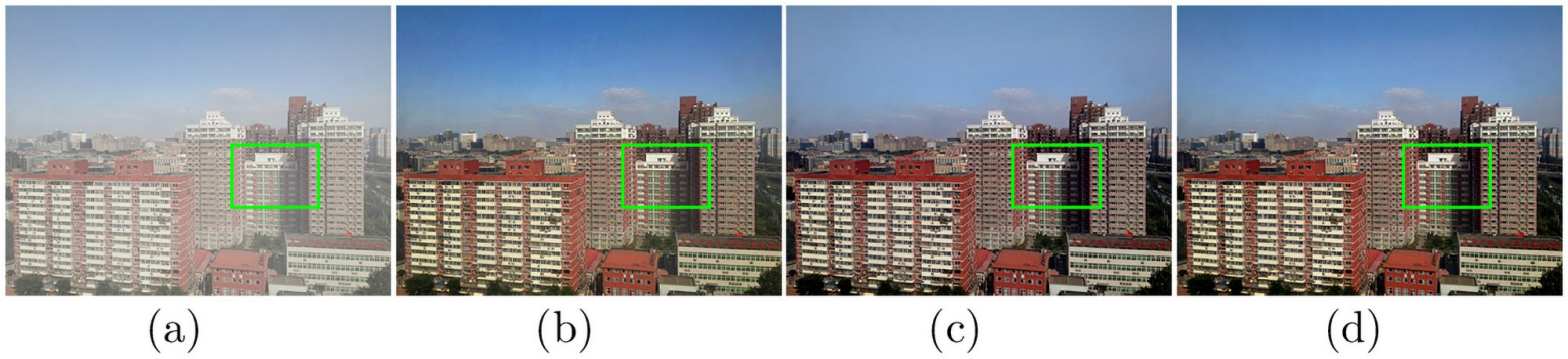
Ablation studies: Comparison of the results on iterative optimization, learning optimization, the proposed method. (a)Hazy (b)Iterative optimization (c)Learning optimization (d)Ours.

We conducted an ablation experiment to verify the contribution of the presented model, which combines a physical model-based approach with a learning-based approach. Fig 10 compares the experimental results of dehazing utilizing the numerical iterative model alone, DehazeNet alone, and the mixed model in this paper. The physical properties of the image can be maintained well in the numerical iterative optimization method, but there are still some hazy and dark areas in the restored image. The method of deep learning optimization can effectively eliminate most of the fog but sacrifices color information, as shown in the sky area in the image. The mixed model proposed in this paper effectively integrates the strengths of these two methods and can effectively remove the fog while maintaining the physical properties of the image.

In conclusion, the experimental results show that the presented approach is competitive in dehazing performance compared with other state-of-the-art methods.

## Conclusion

In this paper, a new mixed iterative model is presented to improve the effect of single image dehazing. Noticing that the physical model-based method can yield high fidelity images and that the learning-based method can effectively eliminate haze in the image, we propose a method to fuse the two models and estimate the transmission using DCP and DehazeNet. Hence, our method can retain the physical characteristics of the image and learn the distribution of haze to accurately recover the haze-free image. Furthermore, in order to get more precise results, we also calculate the local atmospheric light in different haze density areas. Finally, by organically integrating the numerical iterative strategy into the proposed method, the joint estimated transmission and atmospheric light are progressively optimized. To prove the generality, our method is evaluated on synthetic and real-world datasets, as well as remote sensing datasets. Thorough experimental results demonstrate that the proposed algorithm can obtain high-quality results quantitatively and qualitatively comparative to the advanced dehazing approaches.
